# Supplementary material for: A network modelling approach to assess non-pharmaceutical disease controls in a worker population: An application to SARS-CoV-2
Source: PLoS Comput Biol. 2021 Jun 16;17(6):e1009058. doi: 10.1371/journal.pcbi.1009058 (PMC8208574; doi:10.1371/journal.pcbi.1009058)
Supplement: Attachment — Submitted filename: review-PCOMPBIOL-D-20-02076.pdf [file pcbi.1009058.s002.pdf]

Review of PCOMPBIOL-D-20-02076

Dear Editor

I've read with interest the ms entitled "*A network modelling approach to assess non-pharmaceutical disease controls in a worker population: An application to SARS-CoV-2*" by Dr Edward M Hill and colleagues. The authors propose and analyze a model aimed at discussing the consequences of "working from home" in times of pandemics – a topic whose importance and timeliness do not require further comments in these months. From the technical perspective. the approach is sound and the results are promising. However, I personally find that, at present, the findings are not very well organized in the ms. In the current version of the manuscript, I see in fact the risk that a general reader can get lost in the multidimensional varieties of analyses, feeling not to be very well guided into a zoo of too- many-panels among too-many-figures. The paper has a good value, though, and I would therefore suggest the authors to find some right metrics that would permit to best valorize its content.

Here are my comments, in order of appearance in the ms, that I share with the only intent of letting the authors clarify some points that I find critical:

ll. 58-59 and 62-63 The proposed model does not account for children and the elderly, and this may be problematic in view of the so-called "variants" that have shown to involve children (and still be risky to the elderly) more than it was expected with the "original" COVID-19. I recognize that this limitation is well credited in the ms (see also Discussion, ll. 436-440), and I'd not ask the authors to incorporate age classes in their model. However, I think it would be important at least to mention (in the discussion?) how the including children and the elderly would change the network approach proposed here. For example, what would be the major difference between modelling "distance learning" for children and "working from home" for adults? The fact that "within each household, members formed fully connected networks" (ll. 61-63) can have major effects on letality, especially for the elderly.

ll. 76-78 and 84-86 It is quite hard to generalize across sectors the idea that contacts with an individual in another workplace is much lower than with individuals in the same workplace (the authors set the probability of the former to 0.05). It seems to me that workers of some sectors (think to "veterinarians" or "postal" or or "cleaning", just to rely on sectors listed in Table 1) are structurally more in contact with people working in other sectors than with colleagues working in their own sector. No?

l. 92 and surroundings Is there any spatial dimension here? What is the territorial scale of interest for the problem at hand? In the authors' example of retail workers and shoppers, it sounds strange to perform a "purely random" (i.e. territorially immaterial) extraction of nodes to create dynamic contacts in the network. There will be "natural" territorial clusters, with consequent spatial effects of disease spread within the network.

ll. 108-109 Unclear, please reformulate.

ll. 113-114 Once again, the territorial/spatial issue seems to become important even here. How in reality can a person meet ”any other person” in the network?

ll. 150-151 I do not think that the model ran by the authors can formally be called a ”SEIR model”. As the authors correctly assume, there is a crucially important pre-symptomatic phase in COVID-19 (l. 154), where patients can be infective even if not yet infectious – a characteristics that is recognized in many models for COVID now, see for example the very recent paper by Du et al. (2021, Lancet Public Health 6:E184), and that was first proposed by Gatto and coauthors (2020, PNAS, 117:10484).

ll. 168-170 and l. 177 To what extent do these assumptions translate into having different transmissibility of infections across different simulations? The combination of two samplings from uniform distributions (within the interval 0.5-0.8 for the probability of an infective individual being asymptomatic and within the interval 0.3-0.7 or the relative infectiousness) sets quite a wide variability of transmission. Is this based on any evidence/reference? Can it be justified?

ll. 190-193 I've checked Ref. 30 and these values were derived from a sample of 379 confirmed COVID-19 cases, so the robustness of such numbers is very low. It is not a serious problem (low robustness might be better than purely speculative assumptions) but I think it would be fair to inform the reader about it.

ll. 195-198 Unclear and seemingly approximative. Could the authors rephrase, please?

subsection ”Proportion of the workforce working from home” I find that the analysis of the effects of sector-based policies, i.e. those requiring people to work from home in different proportions depending on their specific sector, may be extremely important. However, I'm not convinced that the way used by the authors to show their results in the current version is giving it the force it might deserve. I would suggest to re-organize a bit the presentation here (se below).

l. 277 To the best of my understanding, this 70% has been kept constant almost everywhere throughout the study, is that correct? What would be the effect on the recorded dynamics if that proportion would be changed? With the large number of analyses introduced, the most relevant need to be more clearly visible. We know that this specific parameter can assume quite different values in different countries around the world and, although parameterization is here derived in the UK, the results could be of interest for other countries too (as remarked also by the authors later in the paper).

section ”Results” I think that some figures and their presentation in this section do need a deep revision. For example, the paragraph at ll. 296-304 could be expanded (because it is interesting) but should definitely be moved after having let the reader the possibility to explore Figs. 2 and 4, with their important messages. Actually, I would propose the authors to antepone Fig. 4 to Fig. 3, because the ”flexibility” of the model could be understoon and appreciated by our reader only after having given her/him the impression that the model works.

l. 305 I would change the subsection title to ”Synchronous vs asynchronous working schedules” or similar.

l. 309 Where is this shown? It is quite difficult for the reader to understand what is the total effect of having people working from home, rather than having them working in presence (i.e. at workplace), simply because the authors do not evidence enough with an *ad hoc* simulation what would happen in the latter case (i.e., with no policies at all).

l. 311 ”significantly smaller outbreak” – how much smaller? I would be more quantitative, whenever possible. And with a model, the autors can.

l. 317-318 Why should we expect it? Once again, I would suggest the authors to avoid asking the reader to jump here and there through the ms. In doing that, there is a serious risk for the reader to lose the key points of the authors' interesting results!

ll. 323-326 Unclear.

Fig. 2 To be frank, the use of color intensities either to code the fraction of workers working from home (left column) or the days spent at home rather than at the workplace (other columns) makes the figure hardly interpretable. The reader cannot easily discriminate what is the complicated (!) message arising from those many panels. Especially because it is very tempting for her/him to try guessing (through visual comparison only) what are the advantages and disadvantages of different policies. For example, to what extent is the ”Asynchronous” dynamics better than the ”Synchronous” one? Is it possible to quantify the advantage of using one vs the other policy?

Fig. 3 Is there any prior expectation on the approximately linear dependence on the proportion of workers working from home emerging from simulations for the three quantities shown in panels (a), (b) and (c)? Caption: the two-line long series of number is frankly unreadable, while the white markers are invisible. Once again, I suggest the authors to avoid too complicated paths of reading! Not only is the reader invited to watch Fig. 3 at l. 293, when she/he had not yet the time to explore Fig. 2 in full details – see the paragraphs following l. 293. To fully understand what Fig. 3 is about, the reader is also asked to jump reading Table S5. The risk I see here is that ”only the very motivated readers” (i.e. a tiny minority) would follow the authors through such an accidental narrative pathway, don't you think?

ll. 331-332 and Fig. 5 Couldn't the authors find a metric to represent what they very effectively synthetize in words at ll. 331-332, instead of asking the reader to navigate alone among the 12 panels of Fig. 5?

ll. 335-336 and Fig. 6 A similar comment holds for Fig. 6: are 12 panels really needed to simply say the reader that ”The relationship with the proportion of people isolating over time was comparable (Fig. 6)”?

ll. 337-341 and Fig. 7 The wording used to comment Fig. 7a is not ”one-to-one” with the wording used in labels and in the caption. This causes confusion. In particular, should ”trasmission risk scaling” be interpreted as ”reduction on transmission risk” (l. 337)? I would ask the authors to be consistent in wording, so as to help the reader to interpret their results without ambiguity.

Fig. 8 If I understood it correctly, column three of Fig. 8 shows that R_t becomes lower than unity at very similar timings (after almost one month or so) independently of the value of ”Adherence” to the interventions (ranging from 0 to 1). An ordinary reader would naturally ask the authors ”how is that possible”?

ll. 366-371 and Fig. 9 It would be nice to translate these somehow qualitative comments to Fig. 9 into considerations more directly related to the shapes of quantitative findings from that figure. For example, what the concavity/linearity/convexity of the relationships between the various indicators and ”Adherence” has to say about the process under study?

Discussion As a general comment, I think that the discussion/conclusion section of the paper might be improved by incorporating more quantitative considerations that are directly derived from proposed modelling approach. At present, the discussion section is quite qualitative. What is, for example, the benefit in terms of total numbers of infections that cen be obtained by implementing remote working? Another example of relevant question that the authors can answer quantitatively may be ”To what extent the implementation of asynchronous vs synchronous mechanisms of working from remote, with the same fractions of people working from home, can help society to fight consequences of COVID-19?” At present, the discussion section does not permit the reader to grasp sharp conclusions from the authors’ study.

ll. 414-416 Among the many possible sensitivity analyses that one could perform with the proposed model, I think that studying what are the implications of the ”rapidity in the detection of cases” might be of primary importance. Said in another way: should policy makers really invest in rapid detection – which implies costs and care in designing even sophisticated networks of monitoring – or should they better invest in alternative directions?
