# Supplementary material for: A network modelling approach to assess non-pharmaceutical disease controls in a worker population: An application to SARS-CoV-2
Source: PLoS Comput Biol. 2021 Jun 16;17(6):e1009058. doi: 10.1371/journal.pcbi.1009058 (PMC8208574; doi:10.1371/journal.pcbi.1009058)
Supplement: Attachment — Submitted filename: Response to Reviewers.docx [file pcbi.1009058.s003.docx]

**Response to Reviewers**

**A network modelling approach to assess non-pharmaceutical disease controls in a worker population: An application to SARS-CoV-2** by E. M. Hill, B.D. Atkins, M. J. Keeling, L. Dyson & M.J. Tildesley.

*We are grateful for the opportunity to respond to the comments of the Reviewers, which have caused us to improve the manuscript. Herein we provide a detailed response to each suggestion in blue italics interspersed with the original comments in black font.*

*Where appropriate, at the end of a comment we state in red font and within parentheses the line numbers where that edit/addition can be found within the revised manuscript (clean copy and tracked changes copy).*

**Editor**

In light of the reviews, we would like to invite the resubmission of a significantly-revised version that takes into account the reviewers' comments. Both referees agree on the value of the work but they indicated a number of important clarifications and improvements in presentation needed to make the results more accessible to readers. We would like to see these addressed.

***Authors’ response:***

*We are grateful for the time taken by all those involved in reviewing and the editorial process, especially given present circumstances. We find ourselves in agreement with the comments provided.*

*At a broad level, our change to make the results more accessible to readers have included rewriting the Results section text and reducing the number of main manuscript figures in the Results section to five. We have also added new content exploring the sensitivity of the workplace interventions to test, trace and isolate adherence.*

**Reviewer 1**

This paper presents an analysis of epidemic spread on a network of working adults aged 20-70 years — connected by household, social, workplace and ‘other’ links. Workplaces are stratified into 41 sectors defined by ONS data on UK businesses. Degree distributions for work and social layers of the network are inferred from occupation-specific contact data from the Warwick social contact study. The authors explore the impact of 4 different non-pharmaceutical interventions on the final size, peak incidence, epidemic duration and isolation time for transmission parameters chosen to be yield an initial effective reproduction number of approximately 3, appropriate for wild-type SARS-CoV-2. Results demonstrate that increased worker time spent in the home, asynchronous working patterns over a long working week, reducing work team sizes and increased adherence to contact tracing each reduce outbreak size and flatten the epidemic curve, but do not reduce the duration of the outbreak.

The strengths of this analysis are extensive use of workplace and workplace dependent social contact data to parameterise the network, comprehensive exporation of the uncertainties in outcomes due to parameter uncertainties (but see comments below), and the detail with which interventions are implemented in the model (enabling exploration of nuanced policy options). For these reasons I believe this work represents a novel, valuable addition to existing modelling work on non-pharmaceutical interventions.

***Authors’ response:***

*We thank the reviewer for the positive remarks and guiding comments for improving the manuscript. We outline below our revisions to address the issues raised.*

I have a few suggestions that may aid the clarity of the methodology and interpretation:

1) Can the authors comment on the choice of size of the population modelled (10000 nodes). Is this big enough for the workplace network to be representative of the size distribution and social networks across 41 sectors? Or is the reader to understand that it may not be well sampled, and some of the variation in outcomes is driven by workplace size and network realisation?

***Authors’ response:***

*The size of the network was kept small so that it could be reasonably assumed that any node could possibly contact any other node, thus they are all in the same geographical region. We have clarified this in the ‘Simulation outline’ section of the Methods:*

*“The size of the network was kept small due to the assumption that any node could contact any other, thus must be in the same geographical area.”*

(Clean copy: lines 251-252)

(Tracked changes: lines 298-299)

*Some of the variation in outcomes will be a result of network uncertainty. We have added a Supporting Information figure to demonstrate that there is some variability in the workplace size and network realisation between individual simulation replicates. We have also added to the Methods:*

*“When sampling the number of workplaces and individual workplace sizes in each simulation run, there was variation in these distributions, though qualitative features were retained in individual realisations (S1 Text, Fig I).”*

(Clean copy: lines 75-78)

(Tracked changes: lines 83-86)

*There is also considerable variation resulting from the uncertainty in epidemiological parameters. We have sought to convey this variation with a new Supporting Information figure that displays an analogous figure for the work from home intervention, but displays outputs summarised from 20 simulation run on a single network realisation (rather than 1,000 simulations, carried out using 50 distinct network realisations with 20 simulations performed on each network realisation). We have added to the Results section:*

*“We note that there was significant variation in infection and isolation outcomes for each working from home intervention scenario. This was primarily due to the variability in epidemiological factors between simulation runs, such as the distribution of initial infections, the asymptomatic probability, and the relative infectiousness of an asymptomatic case, all of which were randomly generated at the start of each simulation. The different network structures contributed relatively less variation. Results from a collection of simulations performed on a single network realisation also display a large amount of variation (S1 Text, Fig K)”*

(Clean copy: lines 342-349)

(Tracked changes: lines 423-430)

2) How does the choice of network size relate to the choice for the distribution of ‘other’ contacts — is this so that other contacts are in approximately the same geographical region?

***Authors’ response:***

*The distribution for ‘other’ contacts was set so that everyone has an average of 1 'other' contact per day. The whole network is assumed to be in the same geographical region, as it only includes regular contacts. To aid clarity we have added to the Methods:*

*“The entire network was assumed to be contained within the same geographical area, such that any node could possibly be linked with any other node.”*

(Clean copy: lines 51-53)

(Tracked changes: lines 56-57)

3) As mentioned care has been taken to convey the range of possible outcomes given uncertainty in transmission parameters, with simulations repeated for realisations of network and choices of transmission parameters. There are a few parameters that appear may not have be varied. Some of these are noted as subjective choices (e.g. proportion working from home in each sector), and could warrant a sensitivity analysis or further mention in the discussion.

***Authors’ response:***

*We have added three new paragraphs in the Discussion to expand on the possible implications of varying the network, epidemiological and intervention parameters that were fixed.*

(Clean copy: lines 511-538)

(Tracked changes: lines 667-694)

4) Related to the above point — do the authors expect results (particular regarding contact tracing) are sensitive to assumptions regarding the duration of infectiousness of asymptomatic contacts given recent data suggesting it may be the duration of infectiousness rather than absolute infectiousness that differs between asymptomatic and symptomatic cases?

***Authors’ response:***

*An excellent point and it is another valid item that the results may be sensitive to. This also highlights how some uncertainty in the epidemiological process may resolve over time, though our understanding on the role of asymptomatic transmission remains incomplete. This is included as part of the new paragraphs added in response to the previous comment.*

*“As part of our epidemiological parameters, we assumed the absolute infectiousness of an asymptomatic case to be less than a symptomatic case, but the duration of infectiousness to be equal. Recent data suggests that while symptomatic and asymptomatic individuals have similar average peak viral loads and proliferation stage durations, their average duration of clearance stages has been observed to differ. Furthermore, our intervention parameters included a fixed delay in receiving a test result of two days and (for most analyses) a 70% adherence assumption. Our findings may be sensitive to alternative epidemiological model structures and intervention assumptions, with this being a direction of further study.”*

(Clean copy: lines 531-538)

(Tracked changes: lines 687-694)

5) Related to points 1) and 3): as I have understood the paper the underlying degree distribution is for the networks assumed to be fixed, and the model does not explore uncertainty in this. Presumably there is uncertainty in inference of the lognormal distributions for number of contacts, and the possibility that contact patterns have changed in the ~10 years since the adotped contact diary survey was undertaken? It would be good to understand whether these uncertainties are dwarfed by other model uncertainties, or discuss the limitations of assuming a fixed underlying network parameterisation.

***Authors’ response:***

*It is certainly plausible that contact patterns could have altered over the last 10 years (digitalisation of working practices etc). However, we are data-restricted and contact studies with the richness of data to parameterise work sector contacts are, unfortunately, not frequently done. Hence, our perspective is given there is information available to inform contacts (therefore treated as fixed), what are the epidemiological outputs given uncertainty in pathogen characteristics? We now state in the Discussion:*

*“Additionally, we did not explore uncertainty in the underlying degree distributions for the contact networks. Our conjecture would be inclusion of such uncertainty would increase variability in outcomes. The contact distributions were also informed from a single data source, with it possible that contact patterns may have changed in the intervening time since the contact survey was undertaken (approximately 10 years). However, contact studies with the richness of data to parameterise work sector contacts are infrequent, thus we have used the most recent data of the required quality available to us.”*

(Clean copy: lines 517-523)

(Tracked changes: lines 673-679)

6) It appears that all workers are assumed to be full time in the default scenario. Have the authors checked whether assuming a representative proportion of part time workers in the baseline scenario changes theh relative benefit of different interventions?

***Authors’ response:***

*The reviewer is correct that we assumed all workers were full time in our scenarios. We sought broad insights from what we acknowledge is a simplification of the real-world system, but is amenable to analysis. Having part time workers brings additional complexity as there would need to be assumptions on what contact patterns would be on non-workday weekdays. Within the new paragraphs in the Discussion on the implications of parameter sensitivity, we state:*

*“Another item of prospective sensitivity analysis pertaining to network structure is having a representative proportion of part time workers, though this requires additional assumptions on contact patterns during non-workday weekdays and adds complexity to the network generation. Hence, we have presented a pragmatic approach where we have sought broad insights from what we acknowledge is a simplification of a complex real-world system. Using the analysis we performed around worker patterns, we can postulate that replacing a proportion of the full-time working population with asynchronous, part-time workers would result in a reduced outbreak size and severity.”*

(Clean copy: lines 524-530)

(Tracked changes: lines 680-686)

7) Could variation in workplace interactions, proportion able to work from home and proportion isolating when asked, explain local differences in the success of restrictions? Or would this require modelling of other social determinants of risk as mentioned in the discussion?

***Authors’ response:***

*This work has shown that changes in these factors can make a difference to disease transmission and outbreak size. If ‘success’ is defined by the number of infections, then it is certainly possible that these factors could contribute to variation in success between regions, if in fact these factors themselves were shown to vary. However, there are a myriad of other factors to consider and a lack of data to make such conclusions with strong confidence from this work. We have incorporated these remarks into the Discussion:*

*“Our work has shown that changing workplace interactions can make a difference to disease transmission and outbreak size, suggesting that relative effectiveness of these factors could contribute to regional variations in epidemiological outcomes. However, there are characteristics of the underlying contact structure that our model formulation does not presently capture.”*

(Clean copy: lines 540-544)

(Tracked changes: lines 696-700)

**Reviewer 2**

I’ve read with interest the ms entitled “A network modelling approach to assess non-pharmaceutical disease controls in a worker population: An application to SARSCoV-2” by Dr Edward M Hill and colleagues. The authors propose and analyze a model aimed at discussing the consequences of ”working from home” in times of pandemics – a topic whose importance and timeliness do not require further comments in these months. From the technical perspective, the approach is sound and the results are promising. However, I personally find that, at present, the findings are not very well organized in the ms. In the current version of the manuscript, I see in fact the risk that a general reader can get lost in the multidimensional varieties of analyses, feeling not to be very well guided into a zoo of too- many-panels among too-many-figures. The paper has a good value, though, and I would therefore suggest the authors to find some right metrics that would permit to best valorize its content.

***Authors’ response:***

*We are grateful to the reviewer for their considered appraisal of our manuscript. We explain below our changes in response to the fair comments raised, including the reworking of figures with the intent of improving interpretability.*

Here are my comments, in order of appearance in the ms, that I share with the only

intent of letting the authors clarify some points that I find critical:

ll. 58-59 and 62-63 The proposed model does not account for children and the elderly, and this may be problematic in view of the so-called ”variants” that have shown to involve children (and still be risky to the elderly) more than it was expected with the ”original” COVID-19. I recognize that this limitation is well credited in the ms (see also Discussion, ll. 436-440), and I’d not ask the authors to incorporate age classes in their model. However, I think it would be important at least to mention (in the discussion?) how the including children and the elderly would change the network approach proposed here. For example, what would be the major difference between modelling ”distance learning” for children and ”working from home” for adults? The fact that ”within each household, members formed fully connected networks” (ll. 61-63) can have major effects on letality, especially for the elderly.

***Authors’ response:***

*We agree that there would be a need to revise household contact assumptions for households containing multiple generations. We now mention in the Discussion the likely need for the household contact structure for multi-generation households to have a different parameterisation:*

*“Within multi-generational households the relative amount of contact between each generation may differ; our assumption of members of each household forming a fully connected network could be too general in these circumstances, with an alternative parameterisation required. The impact of age-specific interventions on contact structures also requires attention, such as children switching from attending school in-person to online learning (or vice versa).”*

(Clean copy: lines 552-556)

(Tracked changes: lines 709-713)

ll. 76-78 and 84-86 It is quite hard to generalize across sectors the idea that contacts with an individual in another workplace is much lower than with individuals in the same workplace (the authors set the probability of the former to 0.05). It seems to me that workers of some sectors (think to ”veterinarians” or ”postal” or or ”cleaning”, just to rely on sectors listed in Table 1) are structurally more in contact with people working in other sectors than with colleagues working in

their own sector. No?

***Authors’ response:***

*On the remark regarding more contacts with people working in other sectors than with colleagues working in their own sector, we hope that such characteristics would be somewhat captured by the dynamic contact layer. On the generalisation across sectors, this is a fair point. We note that the framework could be parameterised in finer detail, though requires relevant fine-scale data being available. In the absence of such data, we have applied a subjective assumption as a simplification to enable analysis of the system. We have incorporated these details into the Methods:*

*“We subjectively assumed throughout that the probability of making contact with an individual in another workplace, compared to an individual within the same workplace, was 0.05. We applied this consistently across sectors as a simplifying assumption, however, were relevant fine-scale data available then sector-specific parameterisations may be more appropriate.”*

(Clean copy: lines 86-90)

(Tracked changes: lines 94-98)

l. 92 and surroundings Is there any spatial dimension here? What is the territorial scale of interest for the problem at hand? In the authors’ example of retail workers and shoppers, it sounds strange to perform a ”purely random” (i.e. territorially immaterial) extraction of nodes to create dynamic contacts in the network. There will be ”natural” territorial clusters, with consequent spatial effects of disease spread within the network.

***Authors’ response:***

*Given the small size of the network, we considered it reasonable to not explicitly include any particular spatial dimension. However, we do now add the following clarifications in the Methods:*

*“The entire network was assumed to be contained within the same geographical area, such that any node could possibly be linked with any other node.”*

(Clean copy: lines 51-53)

(Tracked changes: lines 56-57)

*“The size of the network was kept small due to the assumption that any node could contact any other, thus must be in the same geographical area.”*

(Clean copy: lines 251-252)

(Tracked changes: lines 298-299)

ll. 108-109 Unclear, please reformulate.

***Authors’ response:***

*We have removed this sentence.*

ll. 113-114 Once again, the territorial/spatial issue seems to become important even here. How in reality can a person meet ”any other person” in the network?

***Authors’ response:***

*We have added detail on our underlying assumption:*

*“We used a fixed daily probability of each individual interacting with any other individual in the network, without clustering or preference. We justify this with the assumption that the entire network is contained within the same geographical area.”*

(Clean copy: lines 117-120)

(Tracked changes: lines 127-130)

ll. 150-151 I do not think that the model ran by the authors can formally be called a ”SEIR model”. As the authors correctly assume, there is a crucially important pre-symptomatic phase in COVID-19 (l. 154), where patients can be infective even if not yet infectious – a characteristics that is recognized in many models for COVID now, see for example the very recent paper by Du et al. (2021, Lancet Public Health 6:E184), and that was first proposed by Gatto and coauthors (2020, PNAS, 117:10484).

***Authors’ response:***

*We have removed the terminology “SEIR” to lessen the chance of it being assumed that our model transmission dynamics are akin to those of the standard SEIR model.*

*“We ran a disease process on the network structure, with each individual being in either a susceptible, latent (infected but not infectious), infectious or recovered state.”*

(Clean copy: lines 154-155)

(Tracked changes: lines 169-171)

ll. 168-170 and l. 177 To what extent do these assumptions translate into having different

transmissibility of infections across different simulations? The combination of two samplings from uniform distributions (within the interval 0.5-0.8 for the probability of an infective individual being asymptomatic and within the interval 0.3-0.7 or the relative infectiousness) sets quite a wide variability of transmission. Is this based on any evidence/reference? Can it be justified?

***Authors’ response:***

*The assessment of these assumptions leading to different transmissibility of infections across different simulations is correct. Our understanding of asymptomatic transmission of SARS-CoV-2 remains incomplete for both these attributes. The distributions we sample and reference (McEvoy2020 & Buitrago-Garcia2020) are what were available at the original time of writing.*

*[McEvoy2020] McEvoy D, McAloon CG, Collins AB, Hunt K, Butler F, et al. The relative infectiousness of asymptomatic SARS-CoV-2 infected persons compared with symptomatic individuals: A rapid scoping review.medRxiv page 2020.07.30.20165084 (2020). doi:10.1101/2020.07.30.2016508*

*[Buitrago-Garcia2020] Buitrago-Garcia D, Egli-Gany D, Counotte MJ, Hossmann S, Imeri H, et al. Occurrence and transmission potential of asymptomatic and presymptomatic SARS-CoV-2 infections: A living systematic review and meta-analysis. PLOS Med. 17 (9):e1003346 (2020). doi:10.1371/journal.pmed.1003346*

*Further, we have added to the Methods that the samples taken from the two distributions were independent.*

*“There remains limited data available to provide a robust quantitative estimate of the relative infectiousness of asymptomatic and symptomatic individuals. However, there are indications that asymptomatic individuals could be less infectious than symptomatic individuals [McEvoy2020,Buitrago-Garcia2020]. Therefore, we assumed that asymptomatic individuals had a lower risk of transmitting infection compared to symptomatic individuals. To reflect the uncertainty in this area, for each simulation we sampled the relative infectiousness of asymptomatics compared to symptomatics from a Uniform(0.3, 0.7) distribution. This was sampled independently to the asymptomatic probability.”*

(Clean copy: lines 172-178)

(Tracked changes: lines 195-204)

ll. 190-193 I’ve checked Ref. 30 and these values were derived from a sample of 379 confirmed COVID-19 cases, so the robustness of such numbers is very low. It is not a serious problem (low robustness might be better than purely speculative assumptions) but I think it would be fair to inform the reader about it.

***Authors’ response:***

*We concur and thank the reviewer for the suggestion. We have extended the paragraph to include both the details on the number of samples and also how that motivated our choice of sampling these parameters from a distribution (thus ensuring the uncertainty in these empirical estimates was propagated).*

*“We highlight that these estimates were made using a sample of 379 confirmed COVID-19 cases, meaning the robustness of the central estimates could be low. As such, we sampled from a distribution to ensure this uncertainty was captured.”*

(Clean copy: lines 191-193)

(Tracked changes: lines 224-226)

ll. 195-198 Unclear and seemingly approximative. Could the authors rephrase, please?

***Authors’ response:***

*Our rephrasing reads:*

*“We calibrated the relative transmission risks to achieve an uncontrolled reproductive number, R_t_, that was, on average, in the range 2-4 for the initial phase of the outbreak. To obtain these early phase transmission dynamics, we applied an equal scaling of 0.8 to all of the transmission risks calculated above”*

(Clean copy: lines 202-205)

(Tracked changes: lines 236-240)

subsection ”Proportion of the workforce working from home” I find that the analysis of the effects of sector-based policies, i.e. those requiring people to work from home in different proportions depending on their specific sector, may be extremely important. However, I’m not convinced that the way used by the authors to show their results in the current version is giving it the force it might

deserve. I would suggest to re-organize a bit the presentation here (se below).

***Authors’ response:***

*We are grateful to the reviewer for this assessment and the subsequent comments on this item. At a higher-level, we have undertaken a rewrite of the results section and organisation of the figures. We respond to the subsequent comments raised by the reviewer in turn.*

l. 277 To the best of my understanding, this 70% has been kept constant almost everywhere throughout the study, is that correct?

***Authors’ response:***

*It is correct that the adherence parameter was set at 70% in the majority of the analysis. We have added to the Methods a description of this parameter:*

*“We used an adherence parameter to capture the proportion of individuals that follow the recommended guidance. This was applied to the isolation and test-and-trace measures, representing the probability that an individual would both adhere to isolation guidance and engage with test and trace. We did not allow partial adherence to one measure and not the other. Adherence to isolation encompassed isolation for any reason: presenting with symptoms themselves, being in the same household as someone displaying symptoms, or being identified as a close contact of an infected individual via contact tracing. Unless otherwise stated, we assumed a constant 70% adherence to isolation and test-and-trace measures.”*

(Clean copy: lines 237-244)

(Tracked changes: lines 283-291)

What would be the effect on the recorded dynamics if that proportion would be changed? With the large number of analyses introduced, the most relevant need to be more clearly visible. We know that this specific parameter can assume quite different values in different countries around the world and, although parameterization is here derived in the UK, the results could be of interest for other countries too (as remarked also by the authors later in the paper).

*We have attempted to address this through our sensitivity analysis with respect to adherence. We show the general effect of changes in adherence and perform the work place interventions in combination with both a higher and lower level of adherence to the test, trace and isolate intervention. We have emphasised the latter in the new layout of results:*

*“Overall, a lack of adherence to underlying isolation and test-and-trace measures led to larger (although shorter) outbreaks and worsened the relative performance of workplace interventions. We obtained qualitatively similar relationships between adherence and the effectiveness of the other workplace targeted interventions considered in this paper (S1 Text, Figs Q-S)”*

(Clean copy: lines 445-448)

(Tracked changes: lines 584-587)

*We have added comment regarding the application of this framework to other locations in the discussion:*

*“The true adherence of the population, and how this could change over time, should be carefully considered when interpreting these results and applying them in other contexts.”*

(Clean copy: lines 496-498)

(Tracked changes: lines 645-647)

section ”Results” I think that some figures and their presentation in this section do need a deep revision. For example, the paragraph at ll. 296-304 could be expanded (because it is interesting) but should definitely be moved after having let the reader the possibility to explore Figs. 2 and 4, with their important messages. Actually, I would propose the authors to antepone Fig. 4 to Fig. 3, because the ”flexibility” of the model could be understood and appreciated by our reader only after having given her/him the impression that the model works.

***Authors’ response:***

*We thank the reviewer for providing comment on the possible reordering of the preexisting figures to help readability. In addition to extensive revisions to the results text, we have cut the number of figures in the results text to five and reduced the displayed metrics to 4 (total infected, peak size, total isolation days and duration):*

*Fig. 2: WFH proportion, 4 panels using main metrics*

*Fig. 3: Worker patterns, 4 panels using main metrics*

*Fig. 4: Covid-secure workplaces, 4 panels using main metrics*

*Fig. 5: Adherence, 4 panels using main metrics*

*Fig. 6: Example of relative impact of change in working practice for different adherence values.*

l. 305 I would change the subsection title to ”Synchronous vs asynchronous working schedules” or similar.

***Authors’ response:***

*We have modified the results subsection title to “Worker patterns” to match the associated title used in the methods.*

(Clean copy: line 350)

(Tracked changes: line 431)

l. 309 Where is this shown? It is quite difficult for the reader to understand what is the total effect of having people working from home, rather than having them working in presence (i.e. at workplace), simply because the authors do not evidence enough with an ad hoc simulation what would happen in the latter case (i.e., with no policies at all).

l. 311 ”significantly smaller outbreak” – how much smaller? I would be more quantitative, whenever possible. And with a model, the authors can.

l. 317-318 Why should we expect it? Once again, I would suggest the authors to avoid asking the reader to jump here and there through the ms. In doing that, there is a serious risk for the reader to lose the key points of the authors’ interesting results!

***Authors’ response:***

*We have rewritten the subsection of the Results that these comments refer to. We apologise for the confusion caused by the lack of clarity on the no intervention policy scenario. Having no policies at all corresponds to all workers working five days per work week (Monday-Friday) at the workplace. We have also integrated quantitative comparisons into the revised text.*

(Clean copy: lines 350-382)

(Tracked changes: lines 431-478)

ll. 323-326 Unclear.

***Authors’ response:***

*We have removed this passage with the intent of focusing the messaging of the paragraph.*

Fig. 2 To be frank, the use of color intensities either to code the fraction of workers working from home (left column) or the days spent at home rather than at the workplace (other columns) makes the figure hardly interpretable. The reader cannot easily discriminate what is the complicated (!) message arising from those many panels. Especially because it is very tempting for her/him to try guessing (through visual comparison only) what are the advantages and disadvantages of

different policies. For example, to what extent is the ”Asynchronous” dynamics better than the ”Synchronous” one? Is it possible to quantify the advantage of using one vs the other policy?

***Authors’ response:***

*We have rearranged our figures and revised the text throughout the Results section. Our amended approach uses the panels in the violin plot figures to convey to the reader the characteristics of the different policies, including the comparison between the synchronous and asynchronous worker pattern policies. Specifically for the temporal figure (originally Figure 2) we have moved it to the Supporting Information where it serves as extra information for the interested reader to explain the witnessed epidemic summary statistics.*

Fig. 3 Is there any prior expectation on the approximately linear dependence on the proportion of workers working from home emerging from simulations for the three quantities shown in panels (a), (b) and (c)? Caption: the two-line long series of number is frankly unreadable, while the white markers are invisible. Once again, I suggest the authors to avoid too complicated paths of reading! Not only is the reader invited to watch Fig. 3 at l. 293, when she/he had not yet the time to explore Fig. 2 in full details – see the paragraphs following l. 293. To fully understand what Fig. 3 is about, the reader is also asked to jump reading Table S5. The risk I see here is that ”only the very motivated readers” (i.e. a tiny minority) would follow the authors through such an accidental narrative pathway, don’t you think?

***Authors’ response:***

*To aid the narrative, what was Figure 3 in the original submission is now Figure 2 (and therefore is the first results plot that the reader sees). For the N-U scenario, we now provide the relevant information on the proportion of each work sector working from home in Table 5. We have replaced the two-line vector of numbers with a reference to Table 5 . We have amended the colours of the violin plots to improve visibility of the white median markers. We have also amended the Results subsection ‘Working from home’.*

(Clean copy: lines 313-349)

(Tracked changes: lines 382-430)

ll. 331-332 and Fig. 5 Couldn’t the authors find a metric to represent what they very effectively synthesize in words at ll. 331-332, instead of asking the reader to navigate alone among the 12 panels of Fig. 5?

ll. 335-336 and Fig. 6 A similar comment holds for Fig. 6: are 12 panels really needed to simply say the reader that ”The relationship with the proportion of people isolating over time was comparable (Fig. 6)”?

***Authors’ response:***

*To condense the text and sharpen the messaging, our primary focus in this subsection is now given to newly made violin plot figures of the outbreak size, peak in infection, isolation time and outbreak duration summary metrics. The temporal figures have been moved to the Supporting Information, becoming an additional item for the interested reader.*

(Clean copy: lines 383-407)

(Tracked changes: lines 479-529)

ll. 337-341 and Fig. 7 The wording used to comment Fig. 7a is not ”one-to-one” with the wording used in labels and in the caption. This causes confusion. In particular, should ”trasmission risk scaling” be interpreted as ”reduction on transmission risk” (l. 337)? I would ask the authors to be consistent in wording, so as to help the reader to interpret their results without ambiguity.

***Authors’ response:***

*We have amended the text in the Results section to make clear how transmission risk scaling and reduction in transmission risk are linked. For example, “a transmission risk scaling of 1 or 0.75 (no reduction in transmission risk or a 25% reduction in transmission risk)”*

Fig. 8 If I understood it correctly, column three of Fig. 8 shows that Rt becomes lower than unity at very similar timings (after almost one month or so) independently of the value of ”Adherence” to the interventions (ranging from 0 to 1). An ordinary reader would naturally ask the authors ”how is that possible”?

***Authors’ response:***

*These outcomes are due to the interplay between decreased transmission at early stages, but greater proportions of susceptibles remaining to infect. This causes the values of Rt at different adherence levels to cross over, though this is not necessarily at unity. We have emphasised this in the results:*

*“These relationships can also be observed in the temporal profiles of infectious cases, isolating individuals and R_t_ (S1 Text, Fig N). Increased adherence resulted in a faster decline in R_t_ at early stages. However, R_t_ remains higher during later stages (but still below 1) due to a greater proportion of the population remaining susceptible, causing the temporal profiles of R_t_ for different adherence levels to cross over. This caused a flatter, longer outbreak, resulting in fewer infections but longer duration. Finally, increased adherence resulted in greater amounts of isolation throughout.”*

(Clean copy: lines 421-426)

(Tracked changes: lines 547-557)

ll. 366-371 and Fig. 9 It would be nice to translate these somehow qualitative comments to Fig. 9 into considerations more directly related to the shapes of quantitative findings from that figure. For example, what the concavity/linearity/convexity of the relationships between the various indicators and ”Adherence” has to say about the process under study?

***Authors’ response:***

*As with the preceding pieces of analysis, we have also overhauled this subsection of the results, seeking to improve the communication of our findings to the reader. These revisions have included elaborating on the data presented in the figures.*

*“We found that increased adherence to isolation and test-and-trace measures resulted in fewer infections overall and a lower peak (Fig. 5(a,b)). From 0% adherence (effectively no NPIs) to 100%, we saw a 50% reduction in overall outbreak size and 75% reduction in infectious prevalence peak size. Nonetheless, whilst increasing adherence introduced greater variability in the epidemic final size (lengthening violin plots with increasing adherence probability), it simultaneously caused a reduction in the variation in the peak in infectious cases (shorter violin plots with increasing adherence probability).”*

(Clean copy: lines 411-417)

(Tracked changes: lines 536-542)

Discussion As a general comment, I think that the discussion/conclusion section of the paper might be improved by incorporating more quantitative considerations that are directly derived from proposed modelling approach. At present, the discussion section is quite qualitative. What is, for example, the benefit in terms of total numbers of infections that can be obtained by implementing remote working? Another example of relevant question that the authors can answer quantitatively may be ”To what extent the implementation of asynchronous vs synchronous mechanisms of working from remote, with the same fractions of people working from home, can help society to fight consequences of COVID-19?” At present, the discussion section does not permit the reader to grasp sharp conclusions from the authors’ study.

***Authors’ response:***

*We include in the Discussion the following quantitative assessments of the benefits in reducing negative epidemiological impacts when the proportion of workers that switch to remote working increases:*

*“Under our modelling assumptions and default parameter values, we found a 60 - 70% decrease in the median estimates of infections, peak infectious prevalence and total isolation-days with everyone working remotely compared to everyone attending the workplace Monday-Friday.”*

(Clean copy: lines 457-460)

(Tracked changes: lines 599-602)

“We observed (under our default parameter set) up to 20% fewer infections and up to a 40% lower infection peak when using an asynchronous work schedule rather than a synchronous work schedule. These differences between worker pattern implementations were most pronounced when fewer (but non-zero) days were spent at the workplace.”

(Clean copy: lines 470-473)

(Tracked changes: lines 617-621)

ll. 414-416 Among the many possible sensitivity analyses that one could perform with the proposed model, I think that studying what are the implications of the ”rapidity in the detection of cases” might be of primary importance. Said in another way: should policy makers really invest in rapid detection – which implies costs and care in designing even sophisticated networks of monitoring – or should they better invest in alternative directions?

***Authors’ response:***

*The reviewer raises a pertinent point in how speed of case detection and investing in monitoring systems to hasten detection is, ultimately, a policy decision. We have integrated this important point to the discussion paragraph on contact tracing:*

*“From the policy maker perspective there are, therefore, trade-offs to consider between investing costs and care in designing sophisticated monitoring networks to enhance rapid detection, or allocating resources to alternative interventions as part of the overall package of infectious disease control measures.”*

(Clean copy: lines 505-508)

(Tracked changes: lines 660-663)
